# Effects of angiotensin receptor–neprilysin inhibition on myocardial energy metabolism and prognosis in patients with acute myocardial infarction complicated by heart failure

**DOI:** 10.3389/fcvm.2025.1550624

**Published:** 2025-08-12

**Authors:** Caiming Cheng, Yu Nie, Di Chen, Yan Yang, Shunji Liang, Qin Yu

**Affiliations:** ^1^ Department of Cardiology, Affiliated Zhongshan Hospital of Dalian University, Dalian, China; ^2^ Department of Geriatrics, No.903 Hospital of PLA Joint Logistics Support Force, Hangzhou, China; ^3^ Zhongshan Clinical College, Dalian University, Dalian, China; ^4^ Graduate School of Dalian Medical University, Dalian, China; ^5^ Department of Echocardiogram, Affiliated Zhongshan Hospital of Dalian University, Dalian, China

**Keywords:** acute myocardial infarction, heart failure, ARNI, MEE, energy metabolism

## Abstract

**Objective:**

This study aims to evaluate the effects of angiotensin receptor–neprilysin inhibitor (ARNI) on myocardial energy metabolism and prognosis in patients with acute myocardial infarction (AMI) complicated by heart failure (HF).

**Methods:**

A retrospective analysis was conducted on data from 244 inpatients admitted to our center, who were diagnosed with AMI complicated by HF. Among these patients, 210 completed a 1-year follow-up. According to the use of angiotensin-converting enzyme inhibitors (ACEI)/angiotensin receptor blockers (ARB)/ARNI, the 210 patients were divided into the ARNI group (107 cases, 51.0%) and the non-ARNI (ACEI/ARB) group (103 cases, 49.0%). The main outcome measures were the changes in myocardial energy expenditure (MEE) and prognostic indicators after 1-year follow-up.

**Results:**

ARNI significantly reduced MEE after 1 year compared with the ACEI/ARB [(129.61 ± 40.81) kcal/min vs. (154.49 ± 47.58) kcal/min, *P* < 0.01]. The MEE level in the HFrEF group was significantly higher than that in the HFmrEF group (*P* < 0.05). The ARNI group showed significantly lower rates of heart failure (23.0% vs. 43.4%, *P* = 0.001), recurrent myocardial infarction (9.8% vs. 22.1%, *P* = 0.009), and renal function deterioration (5.7% vs. 13.1%, *P* = 0.049) than those in the non-ARNI group. ROC analysis identified an MEE (kcal/min) cutoff value of 178, with 85% sensitivity and 64% specificity for the prediction of cardiac death (AUC = 0.74, *P* = 0.007). During the 1-year follow-up, patients with MEE over 178 kcal/min were associated with increased risk of all-cause death compared with those with MEE below 178 kcal/min.

**Conclusion:**

ARNI significantly reduced MEE compared with ACEI/ARB. MEE was significantly associated with the severity of left ventricular systolic dysfunction and long-term prognosis. An MEE value over 178 kcal/min was a powerful predictor of cardiac death and linked with increased risk of 1-year all-cause mortality in patients with AMI complicated by HF.

## 1 Introduction

Heart failure (HF) is a severe and end-stage condition of various cardiovascular diseases, with high rates of disability and mortality (1). Acute myocardial infarction (AMI) is currently one of the most common and significant etiologies of HF globally (2). The occurrence of HF following MI significantly increases the short-term and long-term risks of adverse events and mortality for patients (3). The heart is a highly energy-demanding organ with limited reserves. Therefore, the balanced state of myocardial energy metabolism (MEM) is crucial for sustaining cardiac function, and disturbances in MEM are one of the pathophysiological reasons for the occurrence and development of HF (4). Acute ischemia and hypoxia during AMI lead to MEM disorder which promotes the occurrence and development of HF. Therefore, MEM holds promise as a new target for the treatment of AMI complicated by HF (4–7). Studies have shown that angiotensin receptor–neprilysin inhibitor (ARNI) could reduce the infarct size of the left ventricle after AMI, enhance cardiac function, significantly improve the quality of life of patients, and lower the incidence of major adverse cardiovascular events (MACE) through multiple pathways to improve blood perfusion, thereby significantly improving the short-term and long-term prognosis of AMI patients (8, 9). However, there is still a lack of relevant research on the impact of ARNIs on MEM in patients with AMI complicated by HF. The purpose of this study is to evaluate the impact of ARNI on MEM and prognosis in patients with AMI complicated by HFrEF and HFmrEF, and this research used the angiotensin-converting enzyme inhibitor (ACEI)/ARB treatment group, the standard pharmacological treatment for AMI complicated by heart failure, as the control group. In addition, transthoracic echocardiography was used to measure myocardial energy expenditure (MEE).

## 2 Methods

### 2.1 Study subjects and grouping

A retrospective analysis was conducted on data from 244 inpatients admitted to the Department of Cardiology, Affiliated Zhongshan Hospital of Dalian University from February 2018 to February 2022. All patients were diagnosed with AMI complicated by HFrEF or HFmrEF, and 210 of them completed a 1-year follow-up. The study protocol was approved and carried out in accordance with the recommendations of the Ethics Committee of the Affiliated Zhongshan Hospital of Dalian University and adhered to the international ethical guidelines of the Declaration of Helsinki. All medical data information, images, and other relevant materials from enrolled patients were anonymized by the Medical Information Management Center of the hospital. Based on the medication status of ARNI/ACEI/ARB, the patients were divided into two groups: the ARNI group (107 cases, including 57 cases for HFrEF and 50 cases for HFmrEF) and the non-ARNI group (103 cases, including 50 cases for ACEI and 53 cases for ARB, 37 cases for HFrEF, and 66 cases for HFmrEF). In this study, ACEI/ARB/ARNI therapy was generally initiated within 24–48 h after hemodynamic stabilization following AMI. For patients who had started ACEI in the acute phase, ARNI was introduced after a ≥36 h washout period. The primary outcome measures were the changes in MEE and cardiac function before and after treatment, while the secondary outcome measures were the incidence of cardiovascular death, all-cause death, recurrent myocardial infarction, and readmission due to heart failure after a 1-year follow-up. Drug safety indicators included symptomatic hypotension, deterioration of renal function, hyperkalemia, angioedema, and dry cough.

### 2.2 Inclusion criteria

(1) Age > 18 years old; (2) history of AMI (diagnosed according to globally unified criteria) (≥30 days); (3) AMI complicated by HF meeting the diagnostic criteria for HFrEF and HFmrEF outlined in the “2020 Expert Consensus on Prevention and Treatment of Post-Myocardial Infarction Heart Failure” (3). (4) hemodynamically stable before initiating ARNI or non-ARNI treatment, with a systolic blood pressure (BP) not less than 90 mmHg within 6 h prior to ARNI initiation; (5) Exclusion of heart failure caused by other etiologies.

### 2.3 Exclusion criteria

(1) Intolerance to ACEI/ARB/ARNI; (2) presence of left ventricular aneurysm, pulmonary heart disease, severe hepatic or renal dysfunction, severe infections, malignancy, hematological or autoimmune diseases, or abnormal thyroid function; (3) use of medications that affect MEM (such as trimetazidine, coenzyme Q10, levocarnitine, and phosphocreatine); (4) lost to follow-up. Discontinuation criteria during treatment: severe renal dysfunction (serum creatinine ≥265 umol/L), hyperkalemia (serum potassium ≥5.5 mmol/L), symptomatic hypotension with systolic blood pressure <90 mmHg.

### 2.4 General data collection

Patient clinical data of selected individuals were collected from the Dragon and Jiahe Electronic Medical Record (EMR) systems. The data primarily included patient age, gender, BMI, New York Heart Association (NYHA) functional classification of heart failure, blood pressure (BP), heart rate (HR), comorbidities, echocardiographic findings, and biochemical indexes.

**Figure 1:**
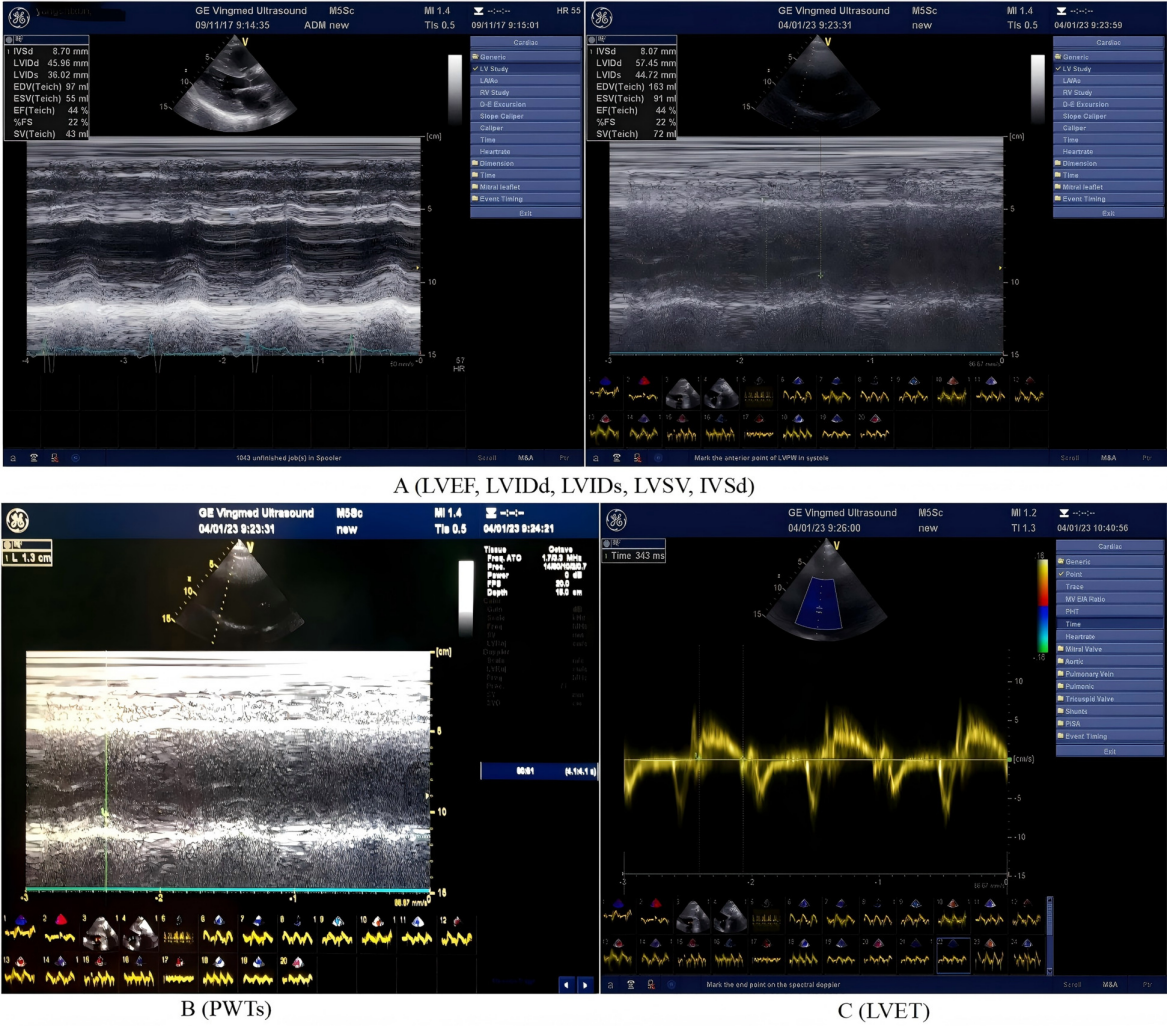
Echocardiographic parameters (including left ventricular structural, functional, volumetric and ejection time) and measurement methods. The panel A (LVEF, LVIDd, LVIDs, LVSV, and IVSd): LVEF, left ventricular ejection fraction; LVIDd, left ventricular internal diameter in diastole; LVIDs, left ventricular internal dimension in systole; LVSV, left ventricular stroke volume; IVSd, interventricular septal thickness in diastole. The panel B (PWTs): left ventricular posterior wall systolic thickness. The panel C (LVET): left ventricular ejection time.

As shown in Figure 1, the PHILIPS EPIQ7 ultrasound system was used for cardiac ultrasound examination, with frequency set to 2–4 MHz. The heart was measured under a standard parasternal long-axis section. The patient took the left lying position to measure the left ventricular ejection fraction (LVEF), peak blood flow (E) in early mitral valve diastole, early mitral annulus velocity (e′), stroke volume (SV), tricuspid regurgitation velocity (TRvelocity), and E/e′. Additional measurements included ventricular septal thickness (IVSD), left ventricular posterior wall systolic thickness (PWTs), left ventricular end-systolic diameter (LVIDs), and end-diastolic diameter (LVIDd). Aortic valve ejection time (ET) was measured by Doppler blood flow spectrum. Each index was continuously measured for three cardiac cycles, and the average value was taken. Left ventricular end-systolic peripheral wall stress (cESS) was calculated, and MEE was calculated using the following formula (10, 11):

$$\begin{array}{c} \mathrm{cESS}=\frac{\mathrm{SBP}\times (\mathrm{LVIDS}/2{)}^{2}\;\times \left\{1+\frac{{(\mathrm{LVIDs}/2+\mathrm{PWTs})}^{2}}{{(\mathrm{LVIDs}/2+\mathrm{PWTs}/2)}^{2}}\right\}}{{(\mathrm{LVIDs}/2+\mathrm{PWTs})}^{2}\text{-}(\mathrm{LVIDs}/2{)}^{2}}\\ \mathrm{MEE}(\mathrm{kcal}/\min)=\mathrm{MEE}(\mathrm{kcal}/\mathrm{systole})\;\times \mathrm{HR}=\mathrm{cESS}\times \mathrm{LVET}\times \\ \mathrm{LVSV}\times \mathrm{HR}\times 4.2\times 10^{\text{-}4} \end{array}$$

### 2.5 Follow-up methods

All enrolled patients underwent on-site follow-up at 6 and 12 months. The observed indicators are as follows:

1. Baseline (data collection at the baseline was performed before the ARNI/ACEI/ARB drug treatment but after percutaneous coronary intervention (PCI) at the time of admission and diagnosis of AMI complicated by HFrEF/HFmrEF): cardiac ultrasound, electrocardiogram, and biochemical markers [including complete blood count, NT-proBNP, cTnI, liver function tests, kidney function tests, electrolytes, fasting blood glucose (FBG), glycated hemoglobin, lipid profile].

2. Six-month follow-up: cardiac ultrasound, electrocardiogram, and biochemical markers, including complete blood count, NT-proBNP, cTnI, liver function tests, kidney function tests, and electrolytes.

3. Twelve-month follow-up: the same assessments were repeated as in the 6-month follow-up.

Major cardiovascular events such as all-cause death, cardiac death, recurrent myocardial infarction, and HF-related rehospitalization were recorded. Drug safety indicators were also monitored, including symptomatic hypotension, renal function deterioration, hyperkalemia, vasovagal edema, and dry cough.

### 2.6 Statistical analysis

Data processing was conducted using SPSS version 26.0 statistical software. Normally distributed continuous variables are expressed as mean ± standard deviation and compared using the *t*-test. Non-normally distributed continuous variables are presented as median and quartiles [M (p25, p75)] and compared using the Mann–Whitney test. The paired Wilcoxon test was used for before-and-after comparisons. Categorical data were expressed as rates or proportions, and the chi-square test or Fisher's exact test was used to compare the two groups. Pearson's analysis method was used for correlation analysis of normal distribution continuous data, Spearman correlation analysis for correlation analysis of non-normal distribution continuous data, and multiple regression analysis to examine the relationship between MEE and various factors. The diagnostic value of indicators was evaluated using ROC curves, with the area under the curve (AUC) indicating diagnostic accuracy. The maximum Youden index was used to determine the optimal diagnostic threshold. Kaplan–Meier curve analysis was used for event-free survival analysis between the two groups. Statistical significance was defined as *P* < 0.05.

## 3 Results

### 3.1 Baseline characteristics

Refer to Table 1 for a comparison of baseline characteristics between the two patient groups, including age, gender, BP, HR, NYHA classification, comorbidities, coronary artery disease risk factors, NT-proBNP, cTNI, Hb, liver and kidney function, electrolytes, lipid profile, FBG, HbA1c, cardiac ultrasound, clinical background medications, and non-pharmacological treatments. There were no statistically significant differences observed (*P* > 0.05).

**Table 1:** Comparison of general data between the two groups.

Parameters	HF ARNI, *n* = 107	HF non-ARNI, *n* = 103 (ACEI, 50 cases/ARB, 53 cases)	*P*
Age, year	69.71 ± 13.16	70.08 ± 13.73	0.843
Male gender (%)	74 (69.1)	77 (74.8)	0.367
ICM (%)	107 (100.0)	103 (100.0)	/
SBP (mmHg)	137.04 ± 19.07	134.42 ± 18.69	0.316
DBP (mmHg)	81.85 ± 13.22	80.55 ± 12.37	0.464
HR (bpm)	80.02 ± 19.24	78.52 ± 15.04	0.532
BMI (kg/m^2^)	25.10 ± 4.50	25.16 ± 3.45	0.911
HFrEF (%)/HFmrEF (%)	57 (53.3)/50 (46.7)	37 (35.9)/66 (64.1)	0.011
NYHA (II–IV)			
NYHA class II (%)	16 (15.0)	27 (26.2)	0.059
NYHA class III (%)	59 (55.1)	49 (47.6)	0.334
NYHA class IV (%)	32 (29.9)	27 (26.2)	0.645
Comorbidities			
HBP (%)	81 (75.7)	77 (74.8)	0.874
DM (%)	56 (52.3)	59 (57.3)	0.472
AF (%)	43 (40.2)	39 (37.9)	0.730
CVD (%)	11 (10.3)	9 (8.7)	0.557
Smoking (%)	45 (42.1)	52 (50.5)	0.221
Laboratory tests			
NT-proBNP (pg/ml)	2,400 (1120, 5899)	2,143 (806, 6070)	0.315
cTNI (ng/L)	0.03 (0.01, 0.08)	0.04 (0.01, 0.08)	0.341
Hb (g/L)	136.32 ± 16.96	131.77 ± 21.97	0.094
ALB (g/L)	37.45 ± 4.66	38.50 ± 5.41	0.131
AST (IU/L)	22 (18, 27)	25 (19, 36)	0.104
ALT (IU/L)	20 (15, 29)	26 (15, 40)	0.053
CREA (umol/L)	78 (66, 95)	82 (68, 96)	0.471
BUN (mmol/L)	7.2 (5.7, 9.0)	7.1 (5.8, 9.2)	0.862
UA (umol/L)	415 (327, 490)	434 (341, 521)	0.556
Na^+^ (mmol/L)	141 (138, 143)	140 (138, 143)	0.532
K^+^ (mmol/L)	4.0 (3.8, 4.3)	4.1 (3.8, 4.4)	0.553
FBG (mmol/L)	6.0 (5.2, 7.7)	6.0 (5.0, 7.5)	0.633
HbA1c (%)	6.5 (5.8, 7.7)	6.3 (5.6, 7.4)	0.104
TG (mmol/L)	1.16 (0.95, 1.57)	1.16 (0.89, 1.81)	0.759
LDL (mmol/L)	2.41 (1.79, 2.98)	2.22 (1.66, 3.17)	0.636
CHO (mmol/L)	4.00 (3.41, 4.87)	4.00 (3.40, 4.85)	0.856
TBIL (umol/L)	14.7 (10.5, 20.3)	13.6 (9.7, 20.1)	0.708
TSH (uTU/ml)	1.64 (1.04, 2.35)	1.62 (1.01, 2.2)	0.835
Echocardiography index			
LVEF (%)	37.90 ± 7.69	39.83 ± 8.21	0.080
E/e′	20.73 ± 8.89	18.62 ± 8.14	0.075
LVIDs (mm)	47 (42, 55)	45 (40, 52)	0.148
LVIDd (mm)	57 (52, 65)	57 (50, 63)	0.599
IVSd (mm)	10 (9, 12)	10 (10, 12)	0.657
LAD (mm)	46 (43, 50)	45 (42, 49)	0.162
MEE (kcal/min)	167.98 ± 45.32	170.51 ± 48.78	0.697
Baseline medical therapy			
Aspirin (%)	71 (65.4)	75 (72.8)	0.309
Clopidogrel (%)	34 (31.8)	38 (36.9)	0.435
Ticagrelor (%)	14 (13.1)	20 (19.4)	0.213
Statin (%)	92 (86.0)	93 (90.3)	0.335
Diuretic (%)	76 (71.0)	71 (68.9)	0.740
Aldosterone (%)	90 (84.1)	80 (77.7)	0.235
Sodium-dependent glucose transporter 2 inhibitor (%)	28 (25.9)	20 (19.0)	0.299
Digoxin (%)	22 (20.6)	12 (11.7)	0.126
β-Blockers (%)	77 (72.6)	74 (71.8)	0.898
Calcium channel blockers (%)	20 (18.7)	26 (25.2)	0.251
Warfarin (%)	5 (4.7)	4 (3.9)	0.778
Rivaroxaban (%)	32 (29.9)	32 (31.1)	0.855
Dabigatran (%)	1 (0.9)	1 (1.0)	0.978
Thrombolytic therapy (%)	1 (0.9)	0 (0.0)	1.0
Percutaneous coronary intervention (%)	86 (80.4)	81 (78.6)	0.756
Coronary artery bypass grafting (%)	7 (6.5)	3 (2.9)	0.333
Conservative treatment (%)	19 (17.8)	21 (20.4)	0.627
Radiofrequency ablation (%)	2 (1.9)	0 (0.0)	0.498
Implantable pacemaker (%)	4 (3.9)	2 (2.0)	0.683
Implantable cardioverter defibrillator (%)	1 (0.9)	1 (1.0)	1.000

ICM, ischemic cardiomyopathy; SBP, systolic blood pressure; DBP, diastolic blood pressure; HR, heart rate; BMI, body mass index; NYHA, New York Heart Association class**;** HBP, hypertension; DM, diabetes; AF, atrial fifibrillation; CVD, cerebrovascular disease; NT-proBNP, N-terminal brain natriuretic peptide precursor; cTNI, cardiac troponin I; Hb, hemoglobin; ALB, albumin; AST, aspartate transaminase; ALT, alanine ransaminase; CREA, serum creatinine; BUN, blood ureanitrogen; UA, uric acid; Na^+^, sodium; K^+^, potassium; FBG, fasting blood glucose; HbA1c, glycosylated hemoglobin, Type A1c; TG, triglyceride; LDL, low-density lipoprotein; CHO, cholesterol; TBIL, total bilirubin; TSH, thyroid-stimulating Hhormone; LVEF, left ventricular ejection fraction; E/e′, peak blood flow (E) in early mitral valve diastole/early mitral annulus velocity (e′); LVIDs, left ventricular internal dimension in systole; LVIDd, left ventricular internal diameter in diastole; IVSd, interventricular septal thickness in diastole; LAD, left atrium diameter; MEE, myocardial energy expenditure.

### 3.2 The impact of ARNI and non-ARNI on MEE, cardiac function, cardiac remodeling, and myocardial injury in patients with AMI complicated by HF

**Figure 2:**
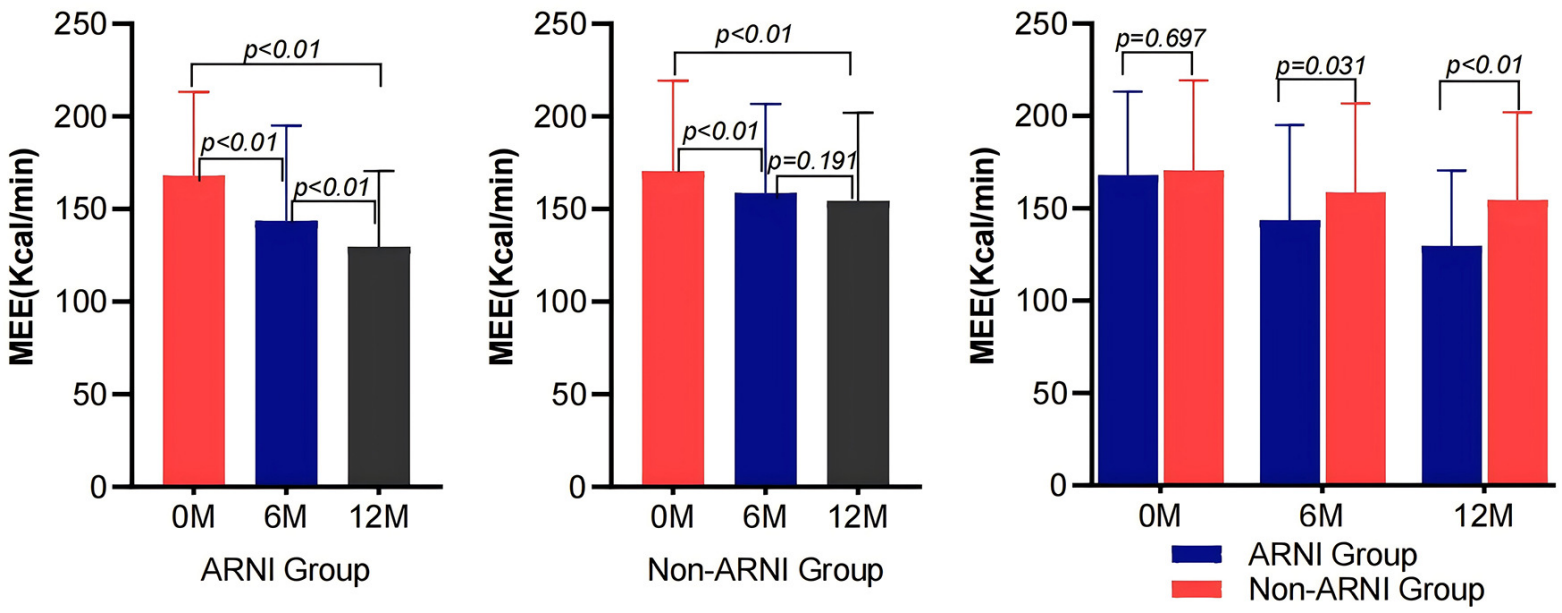
Comparison of MEE between ARNI and non-ARNI before and after treatment.

**Figure 3:**
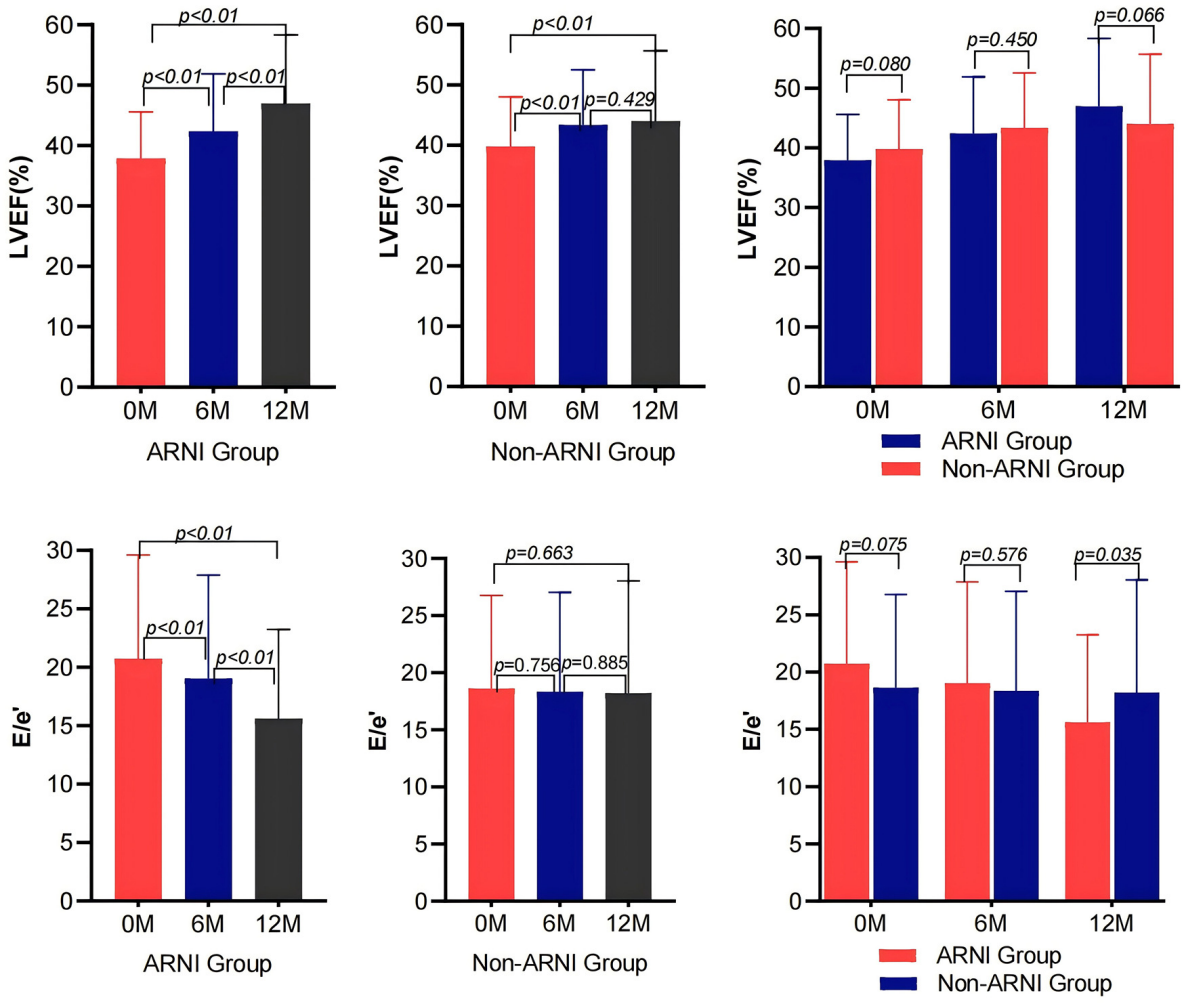
Comparison of LVEF and E/e′ before and after treatment between the ARNI and non-ARNI groups.

As shown in Table 2 and Figure 2, MEE in patients with AMI complicated by HF significantly decreased compared with baseline after treatment in both the ARNI and non-ARNI groups (*P* < 0.05). At the end of the follow-up, MEE in the ARNI group was significantly lower than that in the non-ARNI group (129.61 ± 40.81) kcal/min vs. (154.49 ± 47.58) kcal/min (*P* < 0.01). In both groups, LVEF was significantly increased after treatment compared with baseline in both groups (*P* < 0.05). As indicated in Table 2 and Figure 3, E/e′ in the ARNI group was significantly lower than that in the Non-ARNI group after 12 months of treatment (15.62 ± 7.62 vs. 18.20 ± 9.83, *P* = 0.035).

**Table 2:** Comparison of MEE, cardiac function, myocardial remodeling, myocardial injury markers, and laboratory biochemical parameters between the two groups before and after treatment.

Parameters	ARNI		Non-ARNI	
	Baseline	12M	Baseline	12M
MEE (kcal/min)	167.98 ± 45.32	129.61 ± 40.81*	170.51 ± 48.78	154.49 ± 47.58*^▴^
LVEF (%)	37.90 ± 7.69	44.02 ± 11.65*	39.83 ± 11.21	44.02 ± 11.65*
E/e′	20.73 ± 8.89	15.62 ± 7.62*	18.62 ± 8.14	18.20 ± 9.83^▴^
LVIDs (mm)	47 (42, 55)	40 (35, 49)*	45 (39, 52)	43 (37, 51)*^▴^
LVIDd (mm)	57 (52, 65)	54 (48, 62)*	57 (50, 63)	56 (50, 63)
IVSd (mm)	10 (9, 12)	10 (9, 11)*	10 (10, 12)	10 (9, 12)
LAD (mm)	46 (43, 50)	45 (40, 48)*	45 (42, 49)	44 (40, 48)*
NTpro-BNP (pg/ml)	2,400 (1,120, 5,899)	970 (373, 3,640)*	2,143 (806, 6,070)	1,434 (438, 6,925)*
cTnI (ng/ml)	0.03 (0.01, 0.08)	0.01 (0.01, 0.04)*	0.04 (0.01, 0.08)	0.02 (0.01, 0.06)*^▴^
Hb (g/L)	136.3 ± 17.0	137.7 ± 19.1	131.8 ± 22.0	130.4 ± 22.7^▴^
ALB (g/L)	37.5 ± 4.7	39.3 ± 5.4*	38.5 ± 5.4	39.0 ± 6.1
AST (IU/L)	22 (18, 27)	22 (18, 26)	25 (19, 36)	23 (18, 34)
ALT (IU/L)	20 (15, 29)	19 (14, 24)	26 (15, 40)	22 (14, 30)
CREA (umol/L)	78 (66, 95)	77 (66, 98)	82 (68, 96)	83 (64, 103)
BUN (mmol/L)	7.2 (5.7, 9.0)	6.9 (5.6, 8.6)	7.1 (5.8, 9.2)	7.2 (5.7, 10)
Na^+^ (mmol/L)	141 (138, 143)	141 (138, 142)	140 (138, 143)	140 (138, 142)
K^+^ (mmol/L)	4.0 (3.8, 4.3)	4.2 (3.9, 4.3)	4.1 (3.8, 4.4)	4.1 (3.8, 4.4)

MEE, myocardial energy expenditure; LVEF, left ventricular ejection fraction; E/e′, peak blood flow (E) in early mitral valve diastole/early mitral annulus velocity (e′); LVIDs, left ventricular end-systolic dimension; LVIDd, left ventricular end-diastolic dimension; IVSd, interventricular septal thickness; LAD, left atrium diameter; ARNI, angiotensin receptor–neprilysin inhibitor; non-ARNI (ACEI/ARB), angiotensin-converting enzyme inhibitor/angiotensin II receptor blocker; NTpro-BNP, N-terminal brain natriuretic peptide precursor; cTnI, cardiac troponin I; Hb, hemoglobin; ALB, albumin; AST, aspartate transaminase; ALT, alanine ransaminase; CREA, serum creatinine; BUN, blood ureanitrogen; Na^+^, sodium; K^+^, potassium.

*P* *<* 0.05 indicates statistical significance.

* *P* < 0.05 compared with the baseline within the same group.

^▴^ *P* *<* 0.05 compared with the ARNI group at the same time point. Baseline (0M), 12 months (12M), M (months).

**Figure 4:**
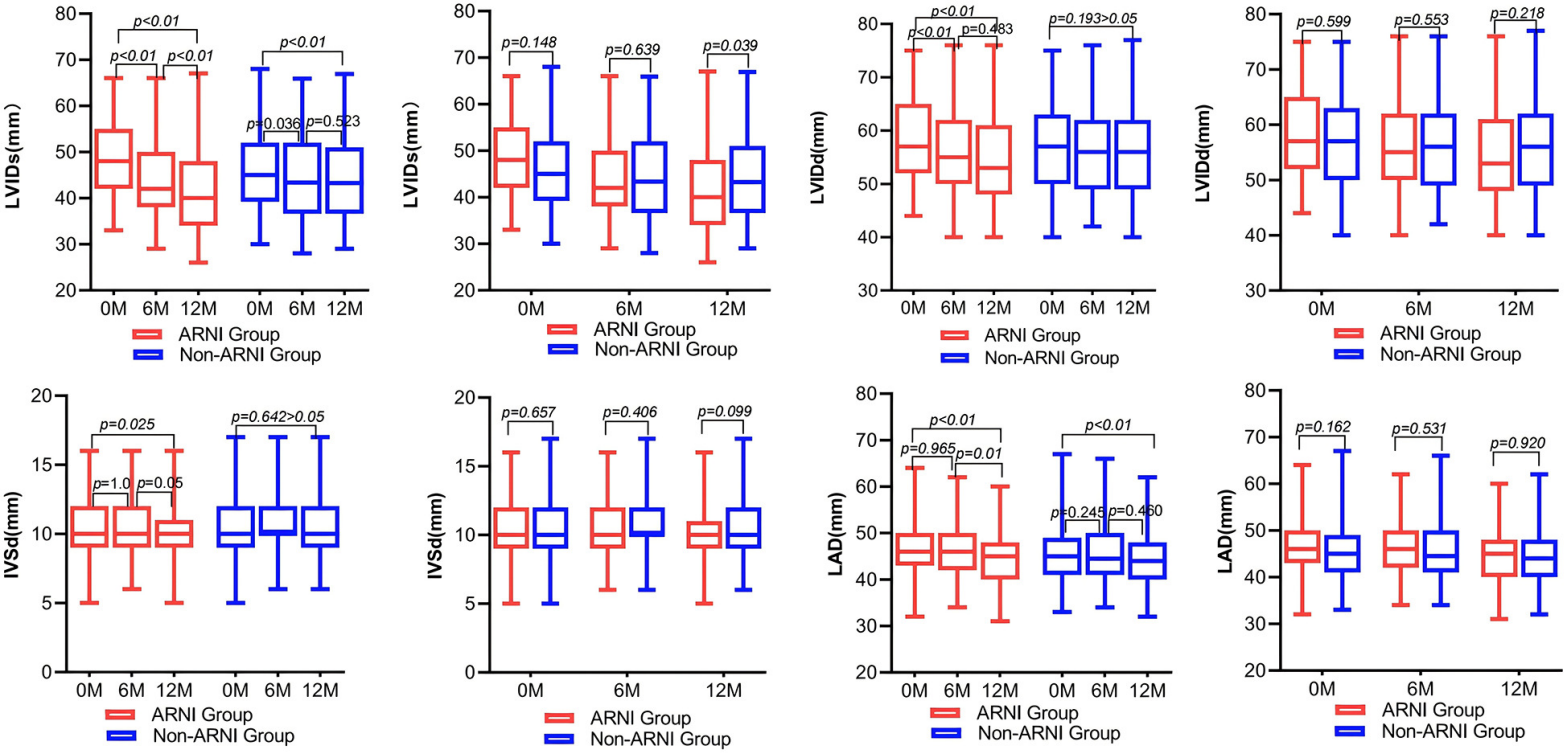
Comparison of left ventricular and left atrial structure between ARNI and non-ARNI before and after treatment.

As shown in Table 2 and Figure 4, AMI complicated by HF patients showed significant reductions in LVIDd, LVIDs, left atrium diameter (LAD), and IVSd in the ARNI group compared with baseline after treatment (*P* < 0.05). In the non-ARNI group, LVIDs and LAD showed a significant decrease compared with baseline after treatment (*P* < 0.05). Although there was a decreasing trend in LVIDd and IVSd after treatment, the differences were not statistically significant (*P* > 0.05). At the end of the follow-up, LVIDs in the ARNI group was significantly lower than that in the non-ARNI group [40 (35, 49) mm vs. 43 (37, 51) mm, *P* = 0.039].

Table 2 showed that in patients with AMI complicated by HF, NT-ProBNP and cTNI significantly decreased compared with baseline after treatment in both groups (*P* < 0.05). At the end of the follow-up, the cTNI levels in the ARNI group were significantly lower than those in the non-ARNI group [0.01 (0.01, 0.04) ng/ml vs. 0.02 (0.01, 0.06) ng/ml, *P* = 0.022], but Hb was higher than that in the non-ARNI group [136.3 ± 17.0 g/L vs. 130.4 ± 22.7 g/L, *P* = 0.012]. Additionally, ALB levels exhibited an increasing trend in the ARNI group and were notably higher than at baseline after treatment in the ARNI group [37.5 ± 4.7 g/L vs. 39.3 ± 5.4 g/L, *P* < 0.01]. There were no significant differences in AST, ALT, CREA, BUN, Na^+^, and K^+^ levels when comparing baseline and posttreatment values or when compared within the same time period between the two groups (*P* *>* 0.05).

### 3.3 Subgroup analysis on the effects of ARNI and non-ARNI on MEE, cardiac function, cardiac remodeling, and myocardial injury in the HFrEF and HFmrEF groups and exploring the impact of cardiac implantable devices on MEE

Tables 3 shows that there were no statistically significant differences in baseline characteristics between the two groups (*P* > 0.05).

**Table 3:** Effects of ARNI and non-ARNI on the change of MEE, cardiac function, cardiac remodeling, and myocardial injury in the HFrEF and HFmrEF groups.

Parameters	ARNI (HFrEF), *n* = 57		Non-ARNI (HFrEF), *n* = 37	
	Baseline	12M	Baseline	12M
LVEF (%)	31.60 ± 4.26	43.42 ± 11.65*	30.46 ± 5.83	36.54 ± 11.34*^▴^
MEE (kcal/min)	182.91 ± 49.27	137.93 ± 44.56*	196.21 ± 46.11	176.43 ± 47.30*^▴^
E/e′	21.67 ± 8.77	16.37 ± 8.93*	21.22 ± 9.12	20.03 ± 8.44^▴^
LVIDs (mm)	54 (48, 60)	44 (38, 51)*	53 (50, 61)	51 (47, 61)*^▴^
LVIDd (mm)	64 (56, 69)	58 (49, 63)*	63 (57, 70)	63 (56, 71)^▴^
PWTs (mm)	13 (12, 14)	12 (11, 14)*	11 (10, 12)	12 (11, 13)
IVSd (mm)	10 (9, 11)	10 (8, 11)*	10 (9, 11)	10 (8, 11)
LAD (mm)	47 (43, 50)	43 (40, 48)*	48 (44, 52)	46 (42, 50)
NTpro-BNP (pg/ml)	3,250 (1,270,7,700)	986 (405, 3,640)*	3,622 (1,550, 8,423)	3,070 (643, 12,563)^▴^
cTnI (ng/ml)	0.03 (0.02, 0.10)	0.01 (0.01, 0.03)*	0.04 (0.03, 0.13)	0.03 (0.01, 0.15)^▴^
Parameters	HFmrEF (*n* = 50)		HFmrEF (*n* = 66)	
LVEF (%)	45.08 ± 2.90	50.98 ± 9.65*	45.08 ± 3.00	48.22 ± 9.58*
MEE (kcal/min)	150.96 ± 33.36	120.12 ± 34.07*	156.11 ± 44.38	148.00 ± 46.28^▴^
E/e′	19.66 ± 8.99	14.76 ± 5.76*	17.17 ± 7.21	17.18 ± 10.45
LVIDs (mm)	42 (37, 44)	38 (33, 41)*	44 (37, 47)	40 (35, 45)^▴^
LVIDd (mm)	55 (49, 58)	51 (47, 60)*	55 (49, 60)	53 (48, 58)
PWTs (mm)	13 (12, 15)	11 (10, 12)*	13 (12, 14)	12 (11, 13)*^▴^
IVSd (mm)	11 (10, 12)	10 (9, 12)*	11 (10, 13)	10 (10, 12)
LAD (mm)	45 (43, 50)	45 (40, 48)*	44 (41, 47)	43 (38, 47)
NTpro-BNP (pg/ml)	1,902 (833, 4,800)	945 (354, 3,540)*	1,305 (652, 3,260)	1,115 (326, 3,130)
cTnI (ng/ml)	0.02 (0.01, 0.05)	0.02 (0.01, 0.04)	0.03 (0.01, 0.07)	0.02 (0.01, 0.04)*

LVEF, left ventricular ejection fraction; MEE, myocardial energy expenditure; E/e′, peak blood flow (E) in early mitral valve diastole/early mitral annulus velocity (e′); LVIDs, left ventricular end-systolic dimension; LVIDd, left ventricular end-diastolic dimension; PWTs, left ventricular posterior wall systolic thickness; IVSd, interventricular septal thickness; LAD, left atrium diameter; NTpro-BNP, N-terminal brain natriuretic peptide precursor; cTnI, cardiac troponin I; ARNI, angiotensin receptor–neprilysin inhibitor; non-ARNI (ACEI/ARB), angiotensin-converting enzyme inhibitor/angiotensin II receptor blocker.

*P* < 0.05 indicates statistical significance.

* *P* < 0.05 compared with the baseline within the same group.

^▴^ *P* < 0.05 compared with the ARNI group at the same time point.

In both the ARNI and non-ARNI groups, LVEF in patients with AMI complicated by HFrEF significantly increased compared with baseline after treatment (*P* < 0.05), and LVEF in the ARNI group was significantly higher than that in the non-ARNI group (43.42 ± 11.65% vs. 36.54 ± 11.34%, *P* *=* 0.006). E/e′ in the ARNI group significantly decreased compared with baseline after treatment (*P* < 0.01) and was also significantly lower than that in the non-ARNI group (16.37 ± 8.93 vs. 20.03 ± 8.44, *P* *=* 0.012). In both groups, MEE in patients with AMI complicated by HFrEF significantly decreased compared with baseline after treatment (*P* < 0.05), and MEE in the ARNI group was significantly lower than that in the non-ARNI group (137.93 ± 44.56 kcal/min vs. 176.43 ± 47.30 kcal/min, *P* < 0.01). In the ARNI group, LVIDd, LVIDs, PWTs, LAD, and IVSd showed significant reductions compared with baseline after treatment (*P* < 0.05). In the non-ARNI group, LVIDs showed a significant decrease compared with baseline after treatment (*P* < 0.05). Although there was a decreasing trend in LVIDd, LAD, and IVSd after treatment, the differences were not statistically significant (*P* > 0.05). At the end of the follow-up, LVIDs [44 (38, 51) mm vs. 51 (47, 61) mm, *P* = 0.004] and LVIDd [58 (49, 63) mm vs. 63 (56, 71) mm, *P* = 0.014] in the ARNI group were significantly lower than those in the non-ARNI group. In the ARNI group, NT-ProBNP and cTNI significantly decreased compared with baseline after treatment (*P* < 0.05), and NT-ProBNP [986 (405, 3,640) pg/ml vs. 3,070 (643, 12,563) pg/ml, *P* < 0.01] and cTNI [0.01 (0.01, 0.03) ng/ml vs. 0.03 (0.01, 0.15) ng/ml, *P* < 0.01] in the ARNI group were significantly lower than those in the non-ARNI group.

In both groups, LVEF in patients with AMI complicated by HFmrEF increased compared with baseline after treatment (*P* < 0.05). E/e′ in the ARNI group significantly decreased compared with baseline after treatment (*P* < 0.01). In the ARNI group, MEE in patients with AMI complicated by HFmrEF significantly decreased compared with baseline after treatment (*P* < 0.01), and MEE in the ARNI group was significantly lower than in the non-ARNI group (120.12 ± 34.07 kcal/min vs. 148.00 ± 46.28 kcal/min, *P* < 0.01). In the ARNI group, LVIDd, LVIDs, PWTs, LAD, and IVSd showed significant reductions compared with baseline after treatment (*P* < 0.05). In the non-ARNI group, PWTs showed a significant decrease compared with baseline after treatment (*P* < 0.01). Although there was a decreasing trend in LVIDd, LVIDs, LAD, and IVSd after treatment, the differences were not statistically significant (*P* > 0.05). At the end of the follow-up, LVIDs [38 (33, 41) mm vs. 40 (35, 45) mm, *P* = 0.036] and PWTs [11 (10, 12) mm vs. 12 (11, 13) mm, *P* < 0.01] in the ARNI group were significantly lower than those in the non-ARNI group. In the ARNI group, NT-ProBNP significantly decreased compared with baseline after treatment (*P* = 0.04), and cTNI significantly decreased in the non-ARNI group compared with baseline after treatment (*P* = 0.03). A comparison of MEE after 1-year follow-up revealed no significant differences among cardiac implantable devices groups (*P* > 0.05) (Table 4).

**Table 4:** The subgroup analysis of MEE.

Variables	Parameters (%)	MEE (kcal/min)	*P*
Percutaneous coronary intervention (PCI)	PCI (79.5%) No PCI (20.5%)	129.73 (109.95, 164.40) 145.48 (119.00, 198.44)	0.081
Coronary artery bypass grafting (CABG)	CABG (4.7%) No CABG (95.3%)	119.81 (97.67, 134.32) 135.84 (112.28, 171.78)	0.133
Radiofrequency ablation (RFA)	RFA (1.0%) No RFA (99.0%)	122.78 (120.55, 125.01) 133.72 (110.76, 171.12)	0.583
Implantable pacemaker (IPM)	IPM (6.6%) No IPM (93.4%)	144.01 (129.73, 183.42) 133.72 (111.78, 171.12)	0.378
Implantable cardioverter defibrillator (ICD)	ICD (1.0%) No ICD (99.0%)	142.30 (120.55, 164.06) 133.07 (110.76, 171.12)	0.815
ARNI Non-ARNI	ARNI (51.0%) Non-ARNI (49.0%)	122.14 (104.16, 146.00) 153.57 (118.57, 184.49)	<0.01

*P* *<* 0.05 indicates statistical significance.ARNI, angiotensin receptor–neprilysin inhibitor; non-ARNI (ACEI/ARB), angiotensin-converting enzyme inhibitor/angiotensin II receptor blocker.

### 3.4 Comparison of clinical endpoints and safety between the ARNI and non-ARNI groups

At the end of the 12-month follow-up, there were 15 cases of all-cause mortality, with 9 in the ARNI group and 6 in the non-ARNI group. Of these, 13 cases were due to cardiovascular death, with 7 in the ARNI group and 6 in the non-ARNI group. The difference in the all-cause mortality rate (7.4% vs. 4.9%, *P* = 0.424) and cardiovascular mortality rate (5.7% vs. 4.9%, *P* = 0.776) was similar between the two groups. The ARNI group had significantly lower rates of hospitalization for heart failure (23.0% vs. 43.4%, *P* *=* 0.001), recurrent myocardial infarction (9.8% vs. 22.1%, *P* = 0.009), and renal function deterioration (5.7% vs. 13.1%, *P* = 0.049) compared with the non-ARNI group. There were no statistically significant differences in the rates of symptomatic hypotension, hyperkalemia, vascular angioedema, and dry cough between the two groups (*P* *>* 0.05).

### 3.5 Clinical correlation between LV dysfunction and MEE

The correlation analysis of baseline MEE and other variables in AMI complicated by HF patients revealed that there was a negative correlation between MEE and LVEF (*r* = −0.481, *P* < 0.01), and MEE showed positive correlations with NYHA classification (*r* = 0.163, *P* = 0.011), LVIDs (*r* = 0.574, *P* < 0.01), LVIDd (*r* = 0.500, *P* < 0.01), LAD (*r* = 0.211, *P* = 0.001), cESS (*r* = 0.595, *P* < 0.01), HR (*r* = 0.239, *P* < 0.01), NT-proBNP (*r* = 0.138, *P* = 0.031), and UA (*r* = 0.135, *P* = 0.035). However, there were no significant correlations between MEE and cTNI, Hb, ALB, AST, ALT, CREA, BUN, Na, K, TG, CHO, LDL-C, TBIL, TSH, FBG, and HbA1c (Table 5). Using MEE as the dependent variable and LVEF, NYHA, LVIDs, LVIDd, LAD, cESS, HR, NT-proBNP, and UA as independent variables, a stepwise multiple linear regression analysis was performed. Table 6 shows that MEE was independently associated with LVIDd (*β* = 0.228, *P* = 0.013), HR (*β* = 0.251, *P* < 0.01), and cESS (*β* = 0.404, *P* < 0.01). Furthermore, as LVIDd, HR, cESS, and MEE increased.

**Table 5:** Correlation analysis between MEE and multiple variables.

Parameters	*r*	*P*	Parameters	*r*	*P*
LVEF	−0.481	<0.01	Hb	0.063	0.329
NYHA	0.163	0.011	ALB	−0.071	0.268
LVIDs	0.574	<0.01	AST	−0.104	0.104
LVIDd	0.500	<0.01	ALT	−0.007	0.915
LAD	0.211	0.001	TBIL	0.110	0.087
E/e′	0.101	0.115	CREA	0.015	0.814
cESS	0.595	<0.01	BUN	0.014	0.829
HR	0.239	<0.01	Na^+^	0.014	0.829
SBP	0.118	0.065	K^+^	−0.006	0.923
DBP	0.098	0.127	FBG	−0.059	0.356
NT-proBNP	0.138	0.031	HAb1c	−0.053	0.441
cTnI	0.123	0.056	TG	−0.001	0.982
UA	0.135	0.035	LDL-C	0.117	0.068
BMI	0.116	0.071	CHO	0.108	0.093
Age	−0.122	0.058	TSH	−0.079	0.218

*P* *<* 0.05 indicates statistical significance.

LVEF, left ventricular ejection fraction; NYHA, New York Heart Association class; LVIDs, left ventricular end-systolic dimension; LVIDd, left ventricular end-diastolic dimension; LAD, left atrium diameter; E/e′, peak blood flow (E) in early mitral valve diastole/early mitral annulus velocity (e′); cESS, circumferential end-systolic wall stress; HR, heart rate; SBP, systolic blood pressure; DBP, diastolic blood pressure; NT-proBNP, N-terminal brain natriuretic peptide precursor; cTNI, cardiac troponin I; UA, uric acid; BMI, body mass index; Hb, hemoglobin; ALB, albumin; AST, aspartate transaminase; ALT, alanine ransaminase; TBIL, total bilirubin; CREA, serum creatinine; BUN, blood ureanitrogen; Na^+^, sodium; K^+^, potassium; FBG, fasting blood glucose; HbA1c, glycosylated hemoglobin, Type A1c; TG, triglyceride; LDL-C, low-density lipoprotein cholesterol; CHO, cholesterol; TSH, thyroid-stimulating hormone.

**Table 6:** Multiple linear stepwise regression analysis of MEE.

Parameters	***β***	SE	*β*	*t*	*P*
LVIDd	1.164	0.466	0.228	2.449	0.013
HR	0.695	0.147	0.251	4.495	<0.01
cESS	0.289	0.057	0.404	5.054	<0.01

*P* *<* 0.05 indicates statistical significance.

LVIDd, left ventricular end-diastolic dimension; HR, heart rate; cESS, circumferential end-systolic wall stress; HR, heart rate.

### 3.6 Predictive ability of MEE for cardiovascular mortality

**Figure 5:**
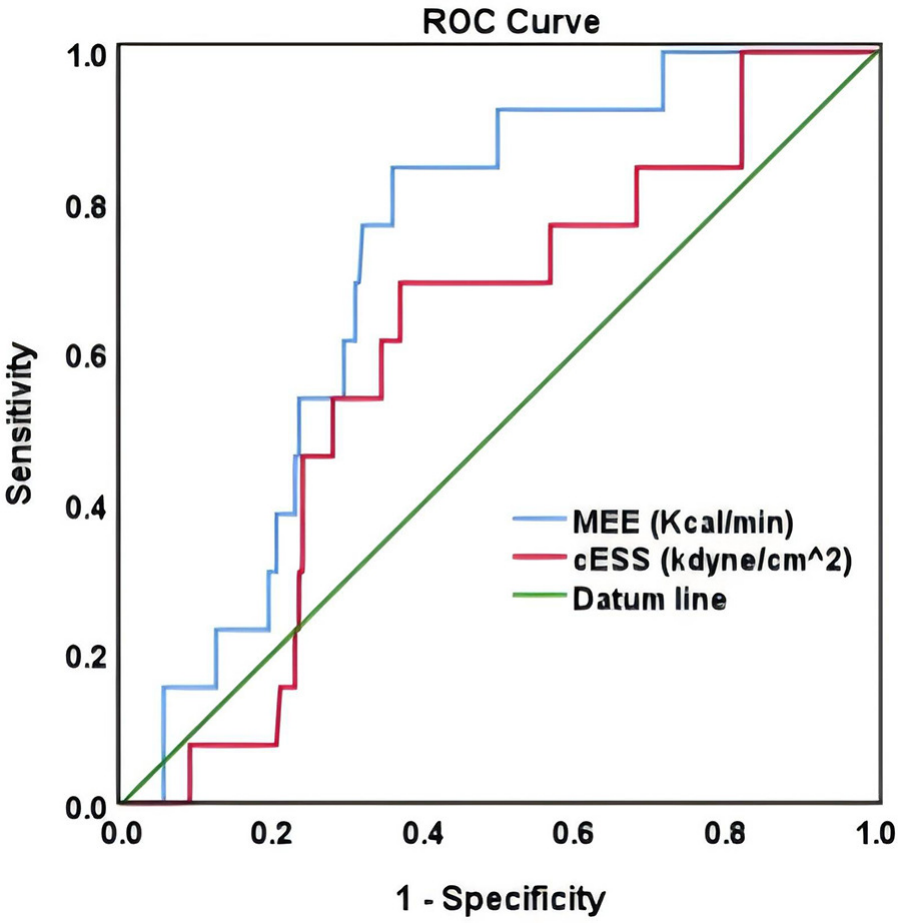
ROC curve of MEE (kcal/min) and cESS (kdyn/cm^2^).

**Figure 6:**
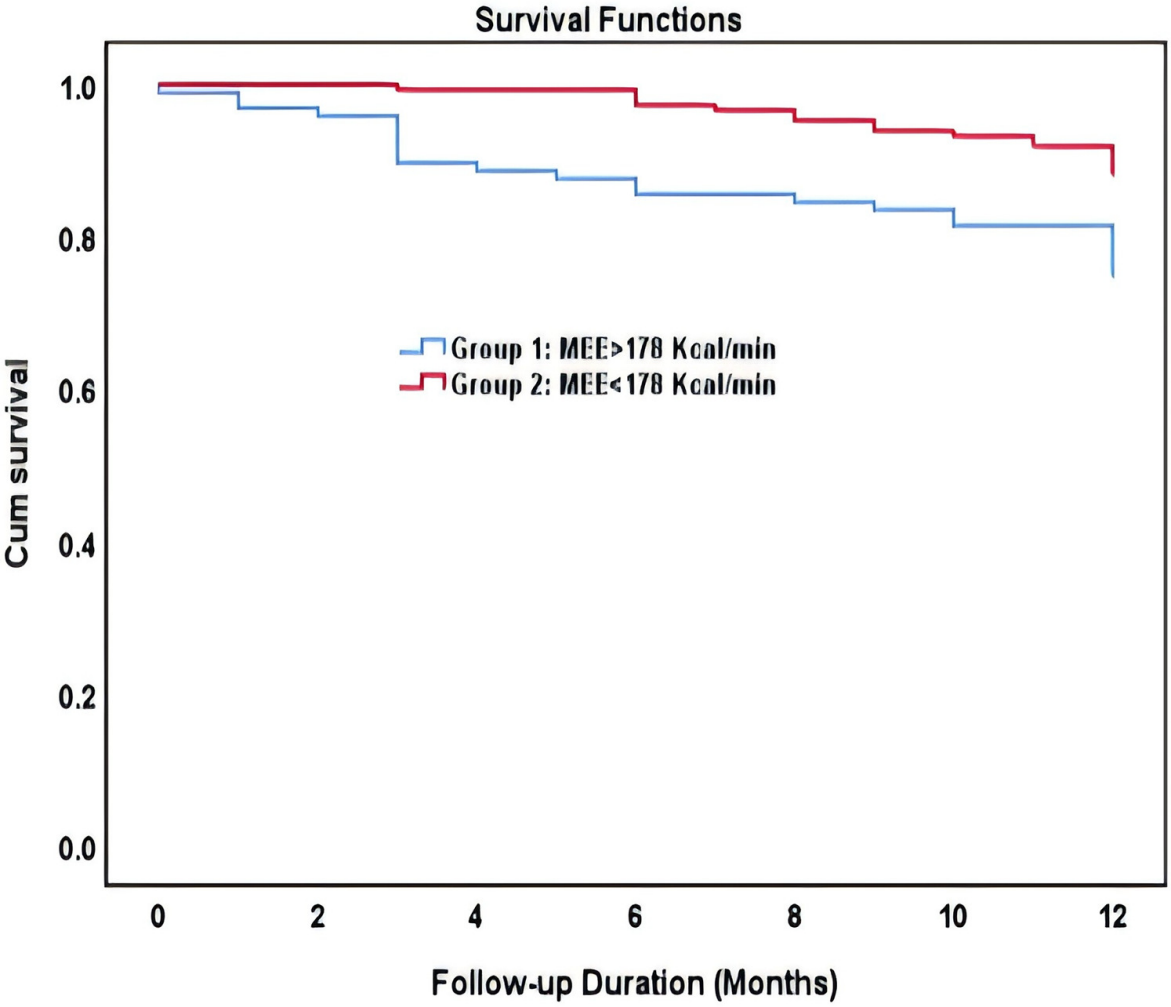
Kaplan–Meier survival curve of participants demonstrating long-term all-cause mortality among patient groups specified based on the myocardial energy expenditure cutoff value of 178 kcal/min.

In ROC analysis, MEE (kcal/min) at a cutoff value of 178 had 85% sensitivity and 64% specificity for prediction of cardiac death (AUC = 0.74, *P* = 0.007) (Table 7; Figure 5). All the patients were divided into Groups 1 and 2 again based on the MEE cutoff value of 178 (Group 1, MEE >178 kcal/min, 97 patients; Group 2, MEE ≤178 kcal/min, 147 patients). Kaplan–Meier analysis according to the long-term event-free survival revealed that the occurrence of events was higher in Group 1 compared with Group 2 (*P* = 0.005) (Figure 6).

**Table 7:** The area under the ROC curve of MEE and cESS.

Parameters	AUC	*P*	Cutoff	Sensitivity (%)	Specificity (%)	SD	95% CI
MEE (kcal/min)	0.74	0.007	178	85	64	0.05	(0.62, 0.83)
cESS (kdyn/cm^2^)	0.61	0.199	252	69	63	0.07	(0.47, 0.74)

*P* < 0.05 indicates statistical significance.

MEE, myocardial energy expenditure; cESS, circumferential end-systolic wall stress; AUC, area under the curve; SD, standard deviation; CI, confidence interval.

## 4 Discussion

The present study showed that in patients with HFmrEF and HFrEF after myocardial infarction, MEE reduction, cardiac functional improvement, reverse remodeling, and mitigation of myocardial injury are more significant in the ARNI group compared with the ACEI/ARB group. The ARNI group has a significantly lower rate of hospitalization for heart failure, recurrent myocardial infarction, and renal function deterioration compared with the ACEI/ARB group. Moreover, MEE (kcal/min) at a cutoff value of 178 could be used as a biomarker to predict cardiac death with 85% sensitivity and 64% specificity (AUC = 0.74), and the occurrence of events was significantly higher in patients with MEE >178kcal/min.

### 4.1 Effects of ARNI on MEE in patients with AMI complicated by HF

Previous studies have demonstrated the benefits of ARNI in cardiac function, ventricular remodeling, myocardial injury, anti-arrhythmias, prognosis, and quality of life of patients with AMI complicated by HF. However, there is limited research regarding the effects of ARNI on myocardial energy metabolism. As part of the GDMT in AMI complicated by HF, the ACEI/ARB treatment group was used as the control group. There are four primary methods available to estimate MEE. Based on safety, simplicity, accuracy, feasibility, cost-effectiveness, stability, and repeatability, transthoracic echocardiography was selected to evaluate MEE in the present study.

This study found that ARNI significantly reduced MEE in patients with AMI complicated by HFrEF and HFmrEF. The known mechanisms underlying the improvement in MEE by ARNI involve its dual blockade of the RASS and neprilysin. It inhibits neprilysin to increase the activity of natriuretic peptides, adrenomedullin, glucagon-like peptide 1 (GLP-1), and skeletal muscle cyclic guanosine monophosphate (cGMP), while simultaneously reducing the activity of dipeptidyl peptidase 4, thus increasing insulin sensitivity (12). Furthermore, ARNI can improve insulin resistance and pancreatic β-cell function and promote glucose metabolism (13). By downregulating NADPH oxidase-1, NADPH oxidase-2, oxidized proteins, γ-H2A histone family member X, and hypoxia-inducible factor 1 alpha and by upregulating Sirtuin-1, Sirtuin-3, superoxide dismutase, catalase, glutathione peroxidase, it alleviates mitochondrial damage induced by oxidative stress and inflammatory response, simultaneously maintain the stability of mitochondrial membrane potential, restore mitochondrial activity, promote autophagy to clear damaged mitochondria, reduce energy consumption, and thus maintain myocardial energy metabolism homeostasis (14–17). Additionally, sacubitril/valsartan dilates blood vessels, reduces both preload and afterload on the heart, decreases myocardial energy consumption, increases blood flow perfusion in ischemic regions, and facilitates the delivery of metabolic substrates (glucose, oxygen), thereby improving myocardial energy metabolism. The further mechanisms and metabolic signaling pathways by which ARNI reduces MEE and improves myocardial energy metabolism still need to be explored and validated through fundamental experiments and clinical research. In this study, ARNI demonstrated a significantly greater improvement in MEE compared with ACEI/ARB, and subgroup analyses revealed that PCI, coronary artery bypass grafting (CABG), radiofrequency ablation (RFA), implantable pacemaker (IPM), and implantable cardioverter defibrillator (ICD) did not have a substantial impact on MEE in patients with AMI complicated by HFrEF and HFmrEF. However, ACEI/ARB also displayed a reduction in MEE during treatment. The reduction in MEE is more pronounced in the first 6 months of medication initiation and gradually diminishes in effectiveness from 6 to 12 months of treatment. The mechanisms underlying this reduction involve the enhanced activity of RASS associated with HF, which can lead to alterations in the insulin and insulin-like growth factor 1 signaling pathway and the generation of reactive oxygen species, resulting in endothelial dysfunction and insulin resistance (18). ACEI can increase fatty acid uptake in HF patients, improve myocardial energy metabolism, and cause inactivation of adrenomedullin, which has a favorable effect on glucose absorption, oxidation, and glycolysis (19). Studies on obese and insulin-resistant animals have shown that ACEI can improve the heart's response to insulin (20).

### 4.2 The impact of ARNI on cardiac function, myocardial remodeling, and myocardial injury in patients with AMI complicated by HF

Sacubitril/valsartan, the first ARNI, exerts cardioprotective effects through dual inhibition of the AT1 receptor of angiotensin II and neprilysin. This dual mechanism attenuates RAAS-mediated vasoconstriction and remodeling while enhancing natriuretic peptide activity, promoting natriuresis, vasodilation, and possessing antifibrotic effects (21, 22).

In this study, the ARNI group showed a significantly stronger inhibition of cardiac remodeling, reduction in MEE and myocardial injury, and improvement in left ventricular systolic and filling compared with the ACEI/ARB group, indicating a more pronounced effect in improving cardiac function. In the PARADISE-MI echocardiographic substudy, at 8-month follow-up, ARNI exhibited a significant advantage over enalapril in inhibiting left ventricular remodeling and reducing left ventricular filling pressure (23). Moreover, in this study, E/e′ in the ARNI group significantly decreased compared with baseline after treatment, and E/e′ in the ARNI group was significantly lower than that in the ACEI/ARB group. Additionally, ARNI is superior to ACEI/ARB in inhibiting myocardial remodeling at the end of follow-up. A multicenter/prospective randomized controlled trial, aimed primarily at observing the improvement of myocardial remodeling in patients with AMI treated with PCI, found that early administration of ARNI effectively prevented cardiac remodeling in AMI patients (24). The SAVE-STEMI trial (25) and a study using an AMI rabbit model (8) both demonstrated that ARNI significantly outperforms enalapril and valsartan in delaying or even reversing left ventricular remodeling and improving systolic function post-MI. Additionally, the latter study found that ARNI was significantly superior to valsartan in reducing post-MI left ventricular infarct area. Another study investigating the therapeutic effects of ARNI in patients with STEMI complicated by HF after emergency PCI found that ARNI significantly delayed ventricular remodeling and improved cardiac function (9). The results of this study are consistent with the aforementioned studies, which demonstrated that ARNI is significantly superior to ACRI/ARB in inhibiting myocardial remodeling, improving cardiac function, and reducing myocardial injury. A study indicated that during the initial week post-high-risk MI, NT-proBNP correlates with the incidence of HF, mortality, and atherosclerotic incidents (26). The PIONEER-HF trial demonstrated that ARNI therapy was superior to ACEI therapy in terms of reducing NT-ProBNP levels among heart failure patients with a diminished ejection fraction during an acute phase (27). Similarly, within this research, NT-proBNP levels significantly decreased in the ARNI group and the ACEI/ARB group in posttreatment, and NT-ProBNP in the ARNI group was significantly lower than in the Non-ARNI group in patients with AMI complicated by HFrEF. A substudy of PARADIGM-HF indicated hemoglobin (Hb) decreased less and the incidence of new anemia was lower with ARNI (28). Correspondingly, at the end of the follow-up of this study, the levels of Hb were found to be higher in the ARNI group compared with the ACEI/ARB groups. The prevalence of liver function abnormalities is common in patients with HFrEF. A substudy of the PARADIGM-HF trial revealed that sacubitril/valsartan improved indicators of liver function compared with enalapril (29). Similarly, ALB levels exhibited a trend of increasing in the ARNI group, which were observed to be higher than at baseline after treatment in this study. The changes in Hb and ALB levels were likely attributed to the ARNI's improvement in cardiac function. Improved cardiac function can enhance systemic organ perfusion, reduce digestive system congestion, and enhance gastrointestinal absorption of proteins and iron supplements which are essential for hematopoiesis, thereby mitigating the decline in Hb. Additionally, the alleviation of hepatic congestion and subsequent reduction in hepatocyte damage thereby contributes to improved liver function and increased synthesis of ALB. In summary, ARNI may improve the overall physiological function in patients with heart failure after myocardial infarction through various indirect or direct mechanisms.

The subanalysis revealed that MEE in the HFrEF group was significantly higher than that in the HFmrEF group in patients with AMI complicated by HF. Several experts have highlighted that MEE shows a significant increase as LVEF decreases and cardiac function grade increases. Moreover, there is a strong correlation between MEE and NT-proBNP levels (30, 31). In this study, MEE showed a significantly negative correlation with LVEF, and MEE showed positive correlations with NYHA classification, NT-proBNP, LVIDs, LVIDd, LAD, cESS, and HR. Multiple regression analysis revealed that MEE was independently associated with LVIDd, HR, and cESS. In the calculation formula for MEE, MEE is directly proportional to HR and cESS. Moreover, reducing HR alleviates myocardial oxygen consumption and decreases MEE. cESS reflects the circumferential stress experienced by the ventricular wall at the end of systole, serving as one of the indices used in cardiac biomechanics to assess myocardial contractility and wall tension (32). Higher cESS indicates that the myocardium requires more energy expenditure and performs more work to overcome ventricular wall stress to maintain normal cardiac output. In addition, this study found that LVIDd had a significant impact on MEE. LVIDd is an indirect indicator for assessing left ventricular preload and myocardial remodeling and is also one of the important parameters for evaluating left ventricular function. Reducing ventricular preload can decrease myocardial work and thus reduce MEE. Reversing myocardial remodeling can improve cardiac function, leading to less energy expenditure under the same conditions of myocardial work. In summary, the results proved that MEE was significantly correlated with the cardiac function, especially LV systolic function, cardiac remodeling, degree of myocardial damage, ventricular wall tension, and ventricular rate. Additionally, the worse the LV systolic function in post-myocardial infarction heart failure patients, the greater the energy expenditure. Namely, the aggravated myocardial oxygen and energy expenditure coexist with enhanced neurohumoral activity, myocardial remodeling, myocardial injury, etc., which may further result in the diminution of myocardial energy storage and deterioration of cardiac function and form the vicious cycle of the above adverse factors in patients with AMI complicated by HF.

At 1-year follow-up, both HFrEF and HFmrEF patients overall had higher LVEF and lower MEE, E/e′, LVIDs, LVIDd, PWTs, IVSd, LAD, NT-proBNP, and cTNI than at baseline, which illustrated that for patients with AMI complicated by HF, cardiac function and MEE can be improved through timely revascularization of the occluded vessel to restore myocardial blood perfusion, long-term standard treatment, and relevant cardiac implantable electronic devices. Furthermore, in the HFrEF group, the change rates for LVEF, NT-proBNP, cTnI, MEE, and cESS were all greater than those in the HFmrEF group. This may reflect that certain treatment regimens have better efficacy in HFrEF than in HFmrEF, particularly ARNI (33). However, in a subset of patients, the LVEF decreased, while MEE, E/e′, LVIDs, LVIDd, PWTs, IVSd, LAD, NT-proBNP, and cTNI increased compared with their baseline, which indicated a persistent deterioration characteristic of LV systolic function in patients with AMI complicated by HFrEF and HFmrEF. Research has demonstrated that any activity resulting in an energy metabolism disorder can precipitate both systolic and diastolic dysfunction of the cardiac mechanics, subsequently triggering ventricular remodeling. The presence of aberrant cardiac energy metabolism is closely related to a decline in cardiac function (34). Reduced MEE might be one mechanism responsible for attenuating myocardial remodeling, relieving myocardial injury and improving cardiac function and prognosis in patients with acute myocardial infarction complicated by heart failure.

### 4.3 The impact of ARNI on the prognosis of patients with AMI complicated by HF

The results of the PARADIGM-HF study showed that ARNI was superior to enalapril in reducing the risk of death from cardiovascular causes or all-cause and hospitalization for heart failure. Additionally, it alleviates symptoms and enhances exercise tolerance in patients with HF (35). The PARADISE-MI study demonstrated that ARNI was not associated with a significantly lower incidence of mortality from cardiovascular causes or incident heart failure than ramipril in patients with AMI complicated by a reduced LVEF, pulmonary congestion, or both (36). However, in subsequent analyses of PARADISE-MI, ARNI was significantly more effective than enalapril in reducing any cause or cardiovascular death and hospitalization for HF. In this study, the ARNI group had a significantly lower rate of hospitalization for heart failure, but there was no significant difference in the rate of cardiovascular mortality and all-cause between the ARNI group and the ACEI/ARB group at the end of the 12-month follow-up, which may be relevant to the small sample size and short observation period of this study. The occurrence rates of adverse events were low in both groups, with no difference in vascular angioedema, hyperkalemia, renal insufficiency, or hepatic dysfunction, and the incidence of hypotension was higher in patients treated with ARNI, while the incidence of dry cough was higher in patients treated with enalapril in the PARADISE-MI study (36–40). In this research regarding the safety profile of ARNI compared with ACEI/ARB, there were no statistically significant differences between the two groups in the occurrence rates of symptomatic hypotension, hyperkalemia, vasomotor edema, or dry cough. Additionally, this study found that ARNI was significantly superior to ACEI/ARB in delaying the deterioration of renal function, which is closely related to its potent effect on improving cardiac function. Improved cardiac function can enhance systemic organ perfusion in heart failure patients, increase renal blood flow, and thus delay the deterioration of renal function. Another study aimed at comparing the cardiovascular outcomes of ARNI and ACEI/ARB in AMI patients found that ARNI was superior to ACEI/ARB in reducing long-term MACE (41). Within this clinical investigation, the ARNI group had a significantly lower rate of hospitalization for heart failure and recurrent myocardial infarction compared with the ACEI/ARB group. Moreover, studies found that 13%–32% of MI patients have left ventricular systolic dysfunction or pulmonary congestion, which is associated with a 2–3 times increased risk of subsequent death or heart failure hospitalization (42). A substudy in PARADISE-MI found that ARNI significantly reduced pulmonary congestion (43), thereby improving prognosis. In conclusion, for patients with AMI complicated by HFrEF and HFmrEF, both ARNI and ACEI/ARB have shown varying degrees of reduction in MEE levels, inhibition or even reversal of myocardial remodeling, improvement in cardiac function, attenuation of myocardial injury, and reduction in MACE, thereby improving prognosis. Moreover, ARNI exhibits superior efficacy over ACEI/ARB in all the aforementioned aspects observed from this study.

In the early and intermediate phases of heart failure, myocardial energy metabolism is relatively normal, while in the terminal phase, the myocardial cells transition from fatty acid metabolism to glucose metabolism as the main source of energy supply (30). However, in the advanced stage, with increased myocardial oxygen and energy consumption, abnormal MEE may reflect the progressive cardiac function deterioration, and MEE may be associated with the prognosis of patients with AMI complicated by HF. Our study provides significant findings on prognostic implications of estimated MEE in relation to LV systolic dysfunction in middle-aged and elderly patients with HFrEF and HFmrEF after myocardial infarction. Increased MEE was correlated with severe LV systolic dysfunction. Furthermore, MEE was a powerful predictor of cardiac death independent of established prognostic factors, including reduced EF, NT-proBNP, cTnI, significant cardiovascular risk factors, comorbidities, and renal function. Stepwise higher rates of cardiac death occurred at MEE values above 178 kcal/min, and MEE over 178 kcal/min is linked with an increased risk of 1-year all-cause mortality in HFrEF and HFmrEF. Similar to the study by Palmieri et al. (10), the present study indicates that depressed EF was associated independently with higher MEE and elevated MEE as an independent predictor of cardiac death in a population-based sample of adults with depressed LV systolic function but without overt congestive heart failure. HF patients generally exhibit a state of cardiac overload. Research indicates that a higher myocardial energy requirement in overloaded hearts is expected based on experimental data on myocardial bioenergetics (44). In patients with AMI complicated with HF, especially those with HFrEF complicated with cardiac overload, increased myocardial energy consumption leads to worsened heart function, thus forming a vicious cycle. The disturbance of myocardial energy metabolism might also be an important factor responsible for the continuous progression of patients with heart failure after myocardial infarction. Therefore, in the treatment of patients with AMI complicated by HF, greater emphasis should be placed on the therapy of myocardial energy metabolism.

## 5 Conclusions

Reduced MEE might be one mechanism responsible for reversing myocardial remodeling, attenuating myocardial injury, elevating cardiac function, lowering MACE, and improving prognosis in patients with AMI complicated by HF. ARNI exhibits superior efficacy over ACEI/ARB in all the aforementioned aspects observed from this study at the end of the 12-month follow-up. MEE is significantly associated with the severity of left ventricular systolic dysfunction and long-term prognosis. MEE is a powerful predictor of cardiac death, and MEE over 178 kcal/min is linked with increased risk of 1-year all-cause mortality in HFrEF and HFmrEF.

### 5.1 Limitations and outlook of this study

Due to the limited study duration, the current analysis is based on the 1-year follow-up data from a single medical center. Further research is needed to initiate a multicenter study with an expanded sample size with follow-up at 1 year and 2 years to better assess the long-term effects of ARNI on MEE, cardiac function, prognosis, and adverse events in patients with post-myocardial infarction heart failure. The calculation formula for MEE used in this study is complex, involving the conversion of mechanical and energetic parameters. However, all measurements in this study were performed by the same experienced cardiac sonographer, minimizing potential confounding factors. In future research protocols, it is recommended to use a variety of methods that can be explored to investigate myocardial energy metabolism while ensuring accuracy. In addition, more studies are needed in the future to clarify the long-term effects of sacubitril/valsartan on myocardial metabolism in patients with post-myocardial infarction heart failure and to explore its long-term effects at the metabolomic level.

## Highlights

The effects of sacubitril/valsartan on myocardial energy metabolism and prognosis in patients with acute myocardial infarction complicated by heart failure are as follows:

1. Previous studies have shown that angiotensin receptor–neprilysin inhibitor (ARNI) can improve and delay ventricular remodeling after AMI. Our research focuses on the impact of ARNI on myocardial energy metabolism in patients with AMI complicated by heart failure.
2. As a kind of non-invasive method to evaluate myocardial energy expenditure (MEE), compared with PET and MRI, echocardiography is simpler, safer, more cost-effective, stable, and repeatable.
3. Compared with GDMT medicine ACEI/ARB, ARNI could reduce MEE, improve myocardial remodeling, and relieve myocardial injury in patients with AMI complicated by heart failure, which improves cardiac function and prognosis.

## Data Availability

The original contributions presented in the study are included in the article/Supplementary Material; further inquiries can be directed to the corresponding author.
